# A Generalized Framework for Quantifying the Dynamics of EEG Event-Related Desynchronization

**DOI:** 10.1371/journal.pcbi.1000453

**Published:** 2009-08-07

**Authors:** Steven Lemm, Klaus-Robert Müller, Gabriel Curio

**Affiliations:** 1 Intelligent Data Analysis Group, Fraunhofer Institute FIRST, Berlin, Germany; 2 Machine Learning Group, Department of Computer Science, Technical University Berlin, Berlin, Germany; 3 Department of Neurology, Campus Benjamin Franklin, Charité University Medicine Berlin, Berlin, Germany; University College London, United Kingdom

## Abstract

Brains were built by evolution to react swiftly to environmental challenges. Thus, sensory stimuli must be processed ad hoc, i.e., independent—to a large extent—from the momentary brain state incidentally prevailing during stimulus occurrence. Accordingly, computational neuroscience strives to model the robust processing of stimuli in the presence of dynamical cortical states. A pivotal feature of ongoing brain activity is the regional predominance of EEG eigenrhythms, such as the occipital alpha or the pericentral mu rhythm, both peaking spectrally at 10 Hz. Here, we establish a novel generalized concept to measure event-related desynchronization (ERD), which allows one to model neural oscillatory dynamics also in the presence of dynamical cortical states. Specifically, we demonstrate that a somatosensory stimulus causes a stereotypic sequence of first an ERD and then an ensuing amplitude overshoot (event-related synchronization), which at a dynamical cortical state becomes evident only if the natural relaxation dynamics of unperturbed EEG rhythms is utilized as reference dynamics. Moreover, this computational approach also encompasses the more general notion of a “conditional ERD,” through which candidate explanatory variables can be scrutinized with regard to their possible impact on a particular oscillatory dynamics under study. Thus, the generalized ERD represents a powerful novel analysis tool for extending our understanding of inter-trial variability of evoked responses and therefore the robust processing of environmental stimuli.

## Author Summary

When Hans Berger described the human EEG in the 1920s, a pivotal finding was the demonstration of prominent oscillations in the frequency range between 8 and 12 Hz, which he called alpha wave rhythm. He also described for the first time the so-called “alpha blockade,” i.e., the suppression of the ongoing alpha activity when the subject opens his eyes. Based on these early findings, induced changes of macroscopic EEG oscillations have been reported for diverse physiological manipulations and processing of sensory information. The magnitude and the latency of these induced changes are, however, subject to variations, even if identical stimuli are processed. In order to enable investigations of the underlying neural mechanisms of these variations, we here establish a mathematical framework which allows one to scrutinize candidate explanatory factors with regard to their possible impact on the characteristics of the induced oscillatory dynamics.

## Introduction

When Hans Berger [1] described the human EEG in the 1920s, a pivotal finding was the demonstration of prominent oscillations in the frequency range between 8 and 12 Hz, which he called alpha wave rhythm. He also described for the first time the so-called “alpha blockade”, i.e., the suppression of the ongoing alpha activity when the subject opens his eyes. In the 1970s Gert Pfurtscheller and colleagues [2] introduced the term event-related desynchronization (ERD) for this kind of frequency specific changes of ongoing EEG activity. Based on these findings induced changes of oscillations have been reported for diverse physiological manipulations and processing of sensory information. For instance voluntary movement results in a circumscribed desynchronization in the upper alpha and lower beta bands, localized close to sensorimotor areas [3],[4]. A desynchronization localized to the auditory cortex following auditory stimuli was reported in MEG recordings [5]. Moreover, the alpha band rhythms demonstrate a relatively widespread desynchronization in perceptual, judgement and memory tasks [6],[7]. In contrast the upper alpha band desynchronization is often topologically restricted, e.g., it develops during the processing of semantic information over the left hemisphere, where the degree of desynchronization is closely linked to semantic memory processes [8]. In addition to oscillations in the alpha and lower beta band, induced oscillations were also reported for the frequency band around 40 Hz with visual stimulation [9] and in movement tasks [10],[11] (for a comprehensive review on ERD cf. [12],[13]).

Beside ERD, EEG correlates of stimulus processing comprise evoked event-related potentials (ERPs); these are commonly assessed by averaging over many instances of stimulus presentations to reduce unrelated EEG activities which can dominate the single-trial responses. To comprehend the interrelationship between evoked and ongoing rhythmic activity various studies have examined the impact of ongoing cortical activity on the latency and the magnitude of ERP components [14]–[19]. Notably, however, the inter-trial variability of ERD itself is not yet fully understood as there exist only a few investigations on the influence of exogenous factors such as stimulus intensity or interstimulus interval on the characteristics of ERD (see, e.g., [20]–[22]) and even less studies on endogenous factors such as attention or the phase and magnitude of EEG rhythms (see, e.g., [23]–[26]). Basically, an adequate data analytical framework for a “state-conditional ERD” is missing which could capture the impact of fluctuating brain states on inter-trial ERD variability. As we will illustrate the customary ERD measure impedes the analysis of state-conditional dependencies of the ERD on endogenous or exogenous factors. Specifically, we will identify the constant baseline, as it is incorporated as reference in the conventional ERD model, as the main cause which hampers a reliable analysis of the ERD variability. In particular, we will show that the use of a constant baseline as reference can lead even to spurious observations of ERD and event-related synchronization (ERS). Based on this result, we generalize the ERD concept by first substituting the constant baseline by a dynamic reference and then derive a reliable measure for state conditional ERD.

To this end the paper is organized as follows: First, we briefly analyze the conventional ERD framework and derive a generalized ERD concept. Second, we extend both, the conventional and the generalized ERD measure towards the analysis of state dependencies. With the application in section “Results” we first comparatively study the capabilities of the two alternative concepts in retrieving known state dependencies by means of artificially generated data. Afterwards, on the basis of a case study, we will outline how our novel framework can be used to investigate the impact of three endogenous factors on the latency and magnitude of the ERD response in the somatosensory system. A discussion along with an outlook concludes the paper.

## Methods

### Ethics statement

One of the authors (SL), who had previous experiences with the acquisition of somatosensory evoked potentials (SEP) as a risk-free routine clinical procedure, served as volunteer subject for the proof-of-concept SEP recording.

### Conventional ERD

To prepare for the introduction of the generalized ERD concept, we first present a brief outline of the conventional ERD measure. The standard measure of ERD quantifies a change in signal band power as difference between a baseline period prior to the event and an post-event period. Typically, the ERD is evaluated as the averaged response over a set of single trials. Up to now, two - basically similar - methods for estimating the ERD have been established, namely the power method [3] and the inter-trial variance method [27]. The advantage of the latter lies in the fact, that it compensates for the spectral bias which is introduced by phase-locked components. However, as the inter-trial variance method requires a slightly more complicated notation, but can be straightforwardly derived from the power method, we will for sake of simplicity introduce the conventional as well as the novel generalized ERD framework solely along the lines of the power method.

**Figure 1:**
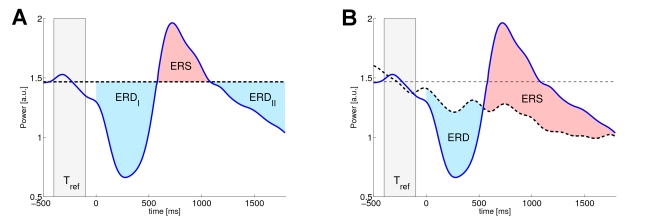
Comparision of conventional and generalized ERD. Conventional ERD (panel A) measures the deviation of the event-related dynamics (blue solid line) from a constant baseline level (black dashed line) that is obtained as averaged power in the reference period 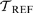. The generalized ERD (gERD), depicted in panel B, defines ERD/S in relation to a dynamic reference (black dashed) which is obtained from catch trials. The reddish and bluish areas of both panels indicate the resulting periods of ERD and ERS, respectively.

In order to attain a mathematical expression of the customary ERD, let 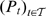 denote the instantaneous signal power in a narrow frequency band during the event-related period 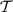. Moreover, let 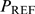 denote the averaged power in the reference period 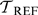, that is
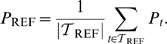
(1)Denoting the expectation value, i.e., the average across trials, by 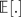, the traditional ERD at time 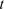 is defined as
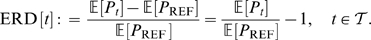
(2)By convention an ERD corresponds to a negative value, i.e., a decrease in power, while ERS refers to an increased signal power [3]. Note that the changes of the signal power are quantified only with respect to the deviation from the fixed, constant baseline level 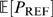. The conventional view on ERD is illustrated in Fig. 1-A.

### Generalized ERD

We start with the following consideration: if an unperturbed dynamics 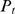 is stationary it follows, that the expectation value 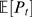 is a constant function and therefore independent of 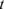. Thus any point in time could be used to empirically estimate this constant value, just by averaging across trials (independent realizations of 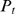). However, if the dynamics 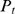 is non-stationary, e.g., exhibits a deterministic negative trend, then the expectation value 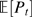 is not necessarily constant and therefore depends on 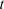.

Consequently, in order to quantify event-related changes of a non-stationary dynamics, an appropriate baseline should reflect the deterministic portion of the unperturbed non-stationarity dynamics. Hence, instead of using a fixed, static reference value, the generalized ERD measure uses the expected unperturbed dynamics as dynamic reference and therefore contrasts the expected dynamics of the instantaneous signal power between an unperturbed and an event-related condition. In order to get a reliable estimate of the expected unperturbed dynamics, we propose the use of so-called *catch trials*, which can be drawn from a continuous EEG measurement during time periods without the occurrence of the event under study (e.g., without somatosensory stimulus or a self-paced movements). This enables us to contrast event-related and reference dynamics directly. Therefore, we define the generalized ERD as the relative difference between both dynamics. Mathematically speaking, let 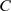 be a binary variable, that distinguishes between the two types of single trials, i.e., between catch 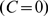 and event-related trials 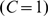. Then we define the *generalized ERD* as
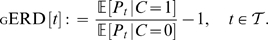
(3)Here 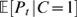 and 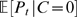 denote the conditional expectation of the band power at time 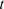 for the event-related and the unperturbed condition, respectively. Complying with the notation of the conventional framework a desynchronization corresponds to negative values, i.e., a decrease in power, while an increase in signal power indicates an event-related synchronization (ERS).

By means of a customized example of somatosensory induced ERD/S Fig. 1 illustrates the two different notions of measuring ERD. In this example the ERD/S is induced at a non-stationary cortical state, that is characterized by a prominent negative drift in the signal band power (readily identifiable from the unperturbed dynamics in the right panel). Consequently, the conventional and the generalized ERD/S yield significantly different results. Most conspicuously, the conventionally measured ERS lasts for a much shorter period and its peak would also be reduced in magnitude. Moreover, relative to the static baseline, the event-related dynamics drops below this level for a second time subsequent to the ERS period. According to the conventional interpretation this would indicate a second ERD phase. However, the cause of this spurious second ERD can be directly attributed to the non-stationary cortical state at stimulus onset. In contrast, the generalized framework which directly compares against the dynamic reference, which captures the deterministic trend, can deal with this phenomenon and yields the familiar ERD-ERS complex.

Note that if the unperturbed dynamics is stationary, then the expected reference dynamics 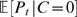 is a constant and is equal to the conventional baseline 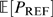. Therefore the conventional and the generalized measure of ERD will coincide with each other in case of analyzing stationary dynamics. In this sense the proposed framework constitutes a generalization of the conventional ERD towards the analysis of spectral perturbations in the presence of dynamical cortical states. Accordingly, the difference between the two approaches only becomes evident when analyzing non-stationary dynamics. One particular field of application of the generalized ERD measure is the analysis of state conditional dependencies of ERD, where the conditional dynamics are not necessarily stationary.

### State conditional ERD

To enable investigations of the influence of arbitrary factors, such as the reaction time in a behavioral response paradigm or the magnitude of a particular EEG eigenrhythm, on the characteristic of the ERD (e.g., the ERD latency or magnitude), we incorporate an additional conditional variable into the ERD measures. To this end, let 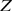 be the explanatory variable representing the factor to be investigated, e.g., representing the level of cortical occipital alpha activity. The conditional gERD, given a particular state 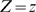 (e.g., low, medium or high level of alpha activity), is defined as
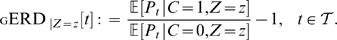
(4)In this formula the denominator and the enumerator represent the state conditional reference and event-related dynamics, respectively. Note, the state variable 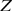 is not necessarily limited to discrete values, such as low, medium and high alpha activity, but can also be continuous valued, e.g., representing the amplitude value itself. For computational aspects of estimating conditional gERD, however, we refer to the **Supplementary Methods section in Text S1**. Moreover, Matlab code is available at http://bbci.de/supplementary/conditionalERD/.

*Remark:* The conventional ERD measure as given in Eqn 2 can be extended in an identical fashion by means of conditional expectations values.
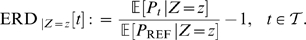
(5)However, in section “Results” will show that this simple extension of the standard measure yields spurious observations of ERD/S. For a detailed description of the empirical estimators of the state conditional ERD please refer to the **Supplementary Methods section in Text S1**.

## Results

The following applications will serve as a proof of concept of the proposed framework. We will illustrate the potential of the proposed *generalized ERD* framework for the analysis of *state conditional ERD* and uncover the limitations of the conventional methods. Initially we conduct a comparative evaluation of both frameworks by means of artificially generated data with known truth. The application in such a controlled, artificial environment will reveal that the *conventional ERD* can give rise to observations of spurious ERD/S. Afterwards we investigate the state dependencies of the characteristics of somatosensory induced ERD on three local cortical states.

### Artificial data

In order to compare the capabilities of the *generalized* and the *conventional conditional ERD* framework properly, we generate three sets of surrogate ERD data that exhibit different kinds of dependency on an explanatory variable 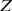. To this end, we use two simple models for the power envelope of unperturbed dynamics on the one hand and for the dampening process on the other hand. Moreover, both models will provide the opportunity to control their dependency on the explanatory factor.

#### Settings

**Figure 2:**
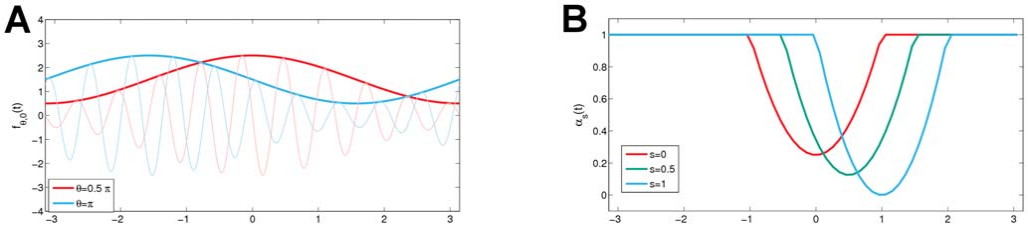
Surrogate data of ongoing activity and the dampening process. Panel A exemplifies two different realizations of the parameterized function 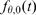, simulating the power envelopes (bold lines) of rhythmic ongoing activity. To establish a better understanding, we also depicted the corresponding oscillations (thin lines) beside the power envelopes. Panel B depicts the multiplicative dampening factor 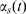 at three different values of the parameter 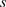.

In particular, we derive the three artificial data sets from a common setup, in which we model the power envelope of the unperturbed ongoing activity as a deterministic, strictly positive function 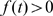, which is construed to capture some essential features of naturally fluctuating EEG oscillations, such as power envelope variability, including short term linear trends (drifts). Specifically, we use the following simple, parameterized model to represent the power envelope of unperturbed rhythmic activity:
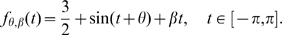
(6)The parameter 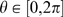 determines the phase of the power envelope, while 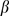 with 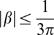 augments it with a distinct linear trend. Later on we will derive different single trial realizations of the unperturbed dynamics by randomly sampling 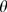 and 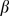 according to a given distribution. However, note that we do not model the oscillations explicitly but merely their envelope. Two different single trial realizations of unperturbed dynamics are exemplified in Fig. 2-A.

To emulate an ERD of the oscillatory process we dampen the power envelope 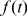 by means of a multiplicative factor 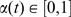. Thus for dampened process 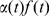 an exhaustive desynchronization corresponds to 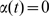, while 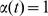 implies no perturbation at all. In order to mimic the valley like shape of ERD we use a simple quadratic function for 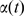 that offers an additional parameter 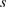 that influences the latency and the magnitude of the attenuation. Specifically, we use the following parameterized function for the dampening 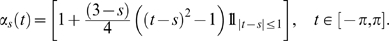
(7)Here 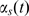 is limited to the interval [0,1] by using the indicator function 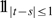 that is equal to 1 for 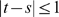 and 0 otherwise. The parameter 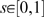 influences the latency and the magnitude of the attenuation and will be linked to the explanatory variable 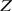 later on. More precisely the maximum attenuation takes place at 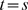, with a maximum dampening factor of 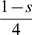. Different realizations of the dampening are exemplified in Fig. 2-B.

Consequently, given a set of parameters 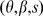, we are able to define the instantaneous power 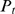 of a single trial separately at the unperturbed and at the event-related condition, such that
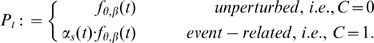
(8)Based on this common architecture Eqn 6–8, we derive three distinct data sets by assigning different probability distribution to the random variables 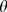, 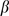 and 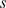 and randomly sampling single trials from these distributions. Moreover, since we are interested in state dependent variations of both, the unperturbed and the dampening process, those distributions will comprise different dependencies on an explanatory variable 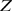. However, for 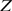 we simply assume a uniform distributed on [0,1], i.e., 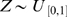. The probabilistic settings and dependencies for the three different data sets are as follows:
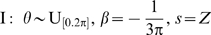
(9)

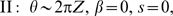
(10)

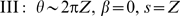
(11)Hence, the first data set solely comprises a dependency of the ERD characteristic on the explanatory variable, i.e., only the dampening process 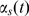 is affected, while the ongoing dynamics 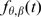 varies independently, but exhibits a deterministic negative trend. In the second example of surrogate ERD data, we implemented a dependency of the initial phasing of the power envelope on the explanatory variable, while the dampening is purely deterministic. The third example of surrogate ERD data is the most complex as it comprises dependencies of both parameters 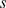 and 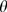 on the explanatory variable 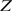. Thus, both, the power envelope of the unperturbed dynamics and the attenuation process depend on the explanatory variable. In order to generate the surrogate ERD data from each model, we repeatedly draw samples for the parameters 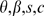 and 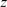, respectively where each sample 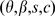 represents a single trial realization of the surrogate ERD data, i.e., each realization of 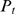, either corresponds (depending on 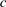) to a catch (unperturbed) or an event-related trial. Needless to say, the estimation of *conditional ERD* requires more data than those of the unconditional. Therefore we sampled 1000 independent single trials 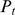,
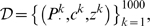
(12)according to the particular settings of each data set. Note that we only observe the single trial data 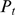 itself along with the state variable 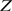 and the binary indicator variable 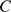. Without loss of generality we generated an equal number of independent single trials per condition, yielding 500 catch trials and 500 event-related trials, respectively.

#### Results

**Figure 3:**
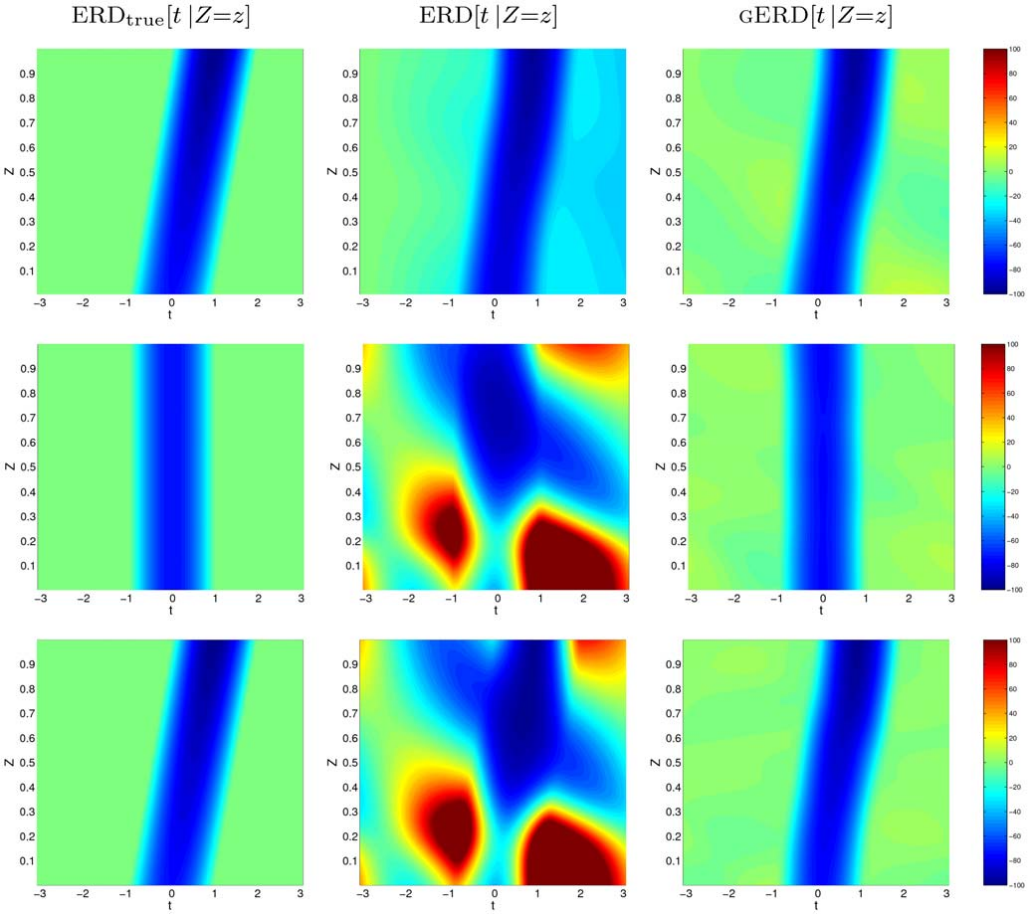
Comparison of both methods for the estimation of state conditional ERD by means of surrugate data. The figure contrasts the true conditional ERD (left column), the estimated conventional conditional ERD (central column) and the estimated generalized conditional ERD (right column). Each row corresponds to a particular artificial data set (I–III, top to bottom). The panels share an identical color coding scheme, where blue and red refer to ERD and ERS, respectively. The vertical and horizontal axes represent the state variable 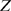 and the time, respectively.

Before comparing the results of the empirical estimators for the conditional ERD, let us begin with some analytical considerations. From the common setup of the artificial datasets we can attain the true conditional ERD as:
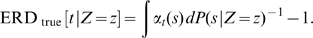
(13)Where 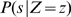 denotes the conditional probability distribution. Moreover, from the definition of the conventional and the generalized conditional ERD (cf. Eqn 4 and 5) and using the instantaneous power at the single time instance 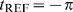 for estimating the static baseline level 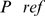 in the conventional framework we obtain:
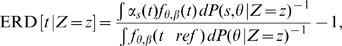
(14)

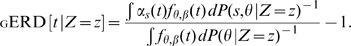
(15)Considering further the particular settings of the three data sets, Table 1 presents the corresponding analytic solutions for the three artificial data sets. So, based on these preceding considerations, we expect the conventional estimator to incorrectly measure the conditional ERD for all three data sets, while the generalized estimator should be capable to retrieve the given underlying functional relationship between the explanatory variable 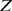 and the ERD dynamics. In Fig. 3 we depict the true conditional ERD and the results of the two competing methods. Comparing the empirical estimates clearly reveals that the *generalized ERD* is capable of recovering the functional dependency of the ERD dynamics on the explanatory variable 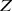, while the conventional estimator miscalculate the conditional ERD and even gives rise to the observation of spurious ERS. On closer examination we can track down the static baseline as the failure cause in the conventional conditional ERD setting. To see this, first note that the conventional setting, using a fixed baseline, implicitly assumes that the expected power of the unperturbed dynamics does not vary with time (weak stationarity). Notably, weak stationarity of the overall distribution does *not* imply weak stationarity of the conditional distributions, which can be easily verified considering the second data set. Here the (unconditional) expectation 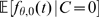, i.e., the average across all catch trials is given as
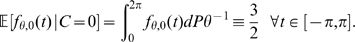
(16)So it is constant and hence weak stationarity is fulfilled. However, conditioning the expectation on the state 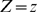 results in
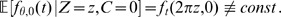
(17)To see this, please note the particular setting 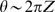 according to Eqn 10. Consequently, the conditional expectation of the unperturbed dynamics is a function of 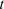, that exhibits a clear non-constant time pattern. Apparently, any constant baseline does not sufficiently represent the intrinsic trends in the unperturbed dynamics. Accordingly, the conventional ERD measure, which relies on the static baseline assumption, incorrectly specifies the conditional unperturbed dynamics and therefore misvalues the true conditional ERD.

**Table 1:** Analytic solutions of conditional ERD for the three artificial datasets.

dataset	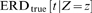	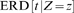	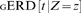
I	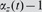	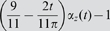	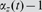
II	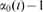	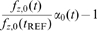	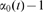
III	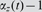	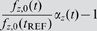	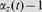

The analytic solutions of the true, the conventional and the generalized conditional ERD for the three artificial datasets according to Eqn 13–15.

### Somatosensory induced desynchronization

The human perirolandic sensorimotor cortices show rhythmic macroscopic EEG/MEG oscillations with spectral peak energies around 10 Hz (localized predominantly over the postcentral somatosensory cortex) and 20 Hz (over the precentral motor cortex) [28]. These so-called 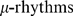 exhibit fast inherent fluctuations as they are limited to brief periods (spindles) of 0.5–2 s duration [29], which appear to occur in the absence of overtly processing sensory information or motor commands. ERD/S of the 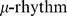 have been reported for different physiological manipulations, e.g., by motor activity, both actual and imagined [3],[30],[31], as well as by somatosensory stimulation [25]. In this context standard trial averages of 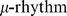 power typically reveal a sequence of attenuation followed by a rebound which often overshoots the pre-event baseline level [31],[32]. In the following we will present a case study, investigating the impact of three endogenous factors on the characteristics of somatosensory induced ERD.

#### Experimental design

**Figure 4:**
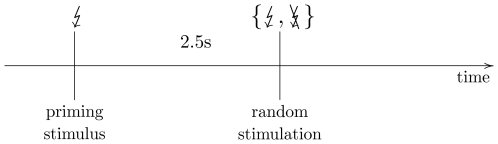
Schematic layout of a single trial. After a first priming stimulus a second stimulus is delivered randomly at a predefined inter-stimulus interval. The responses to the second stimulus are then used for the analysis of conditional ERD.

The brain activity of a healthy subject was recorded using a 64-channel EEG system, at a sampling rate of 1000 Hz. During the experiment the subject was sitting relaxed in a comfortable chair and staring at a fixation cross. ERD of the 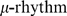 was induced by electrical stimulation of the median nerve at the right wrist. In order to fulfill the requirements imposed by the generalized ERD concept, i.e., to provide *catch trials* for the estimation of the reference dynamics, we deployed a randomized stimulation scheme, which alternates between trial with and without stimulation. In particular, we designed the experimental setting such that each trial consists of a pair of stimulations: a first priming stimulus, always delivered, which is at random followed either by a second stimulation (event-related) or by a void period without stimulation (Fig. 4 depicts a schematic of the single trial setup). The investigations of state conditional ERD are then restricted to the analysis of the responses to the second stimulus only. The priming stimulus in this experimental design mainly serves for setting the somatosensory cortex in a consistently activated, dynamical state, i.e., both, event-related and void periods follow a stimulation for certain. The inter-stimulus interval between the initial priming stimulus and the second randomly delivered stimulus was set to 2.5 seconds. The inter-trial interval (the period between two consecutive initial stimuli) was set to 5 seconds. The intensity of both stimuli was identically set to 10 mA at a pulse width of 0.1 ms , which was slightly below the motor threshold, i.e., the stimuli were not sufficient to evoke a thumb twitch. Using a pseudo-random sequence, we recorded a total of 1200 single trials, i.e., 600 per condition. Restricting the analysis of conditional ERD to the contralateral 10 Hz 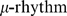, we investigate the impact of three explanatory variables on the magnitude on one hand and the latency of the ERD response on the other. For the three factors we chose the local prestimulus activity of the contralateral 10 Hz 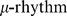 itself, the prestimulus activity of an occipital 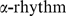 and the magnitude of the ERS response to the priming stimulus. In order to extract the instantaneous signal band power from the occipital and sensorimotor regions, we applied separately optimized spatial and spectral filters, that allow to reduce the cross talk and therewith improve the signal-to-noise ratio (see **Supplementary Methods section in Text S1** for details of the preprocessing). Denoting the extracted instantaneous signal power of the occipital 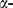 and the contralateral 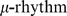 by 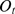 and 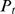, respectively, we define the three explanatory factors as:
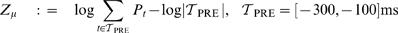
(18)

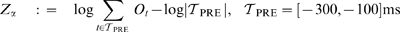
(19)

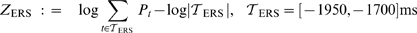
(20)Note that the above intervals are defined relative to the onset of the second, randomly delivered stimulus. Thus, 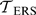 corresponds to the period [550,800]ms relative to the initial priming stimulus, i.e., it covers the initial ERS response. The logarithms in the definition of the explanatory variables are motivated by the fact that the distribution of bandpower is typically similar to a log-normal distribution. Taking the logarithm of the averaged bandpower yields a distribution similar to that of a Gaussian. However, as the logarithm is a monotonic transformation it preserves the neighborhood property of the data and hence does not affect any monotonic relationship between the explanatory variable and the ERD characteristic.

#### Results

**Figure 5:**
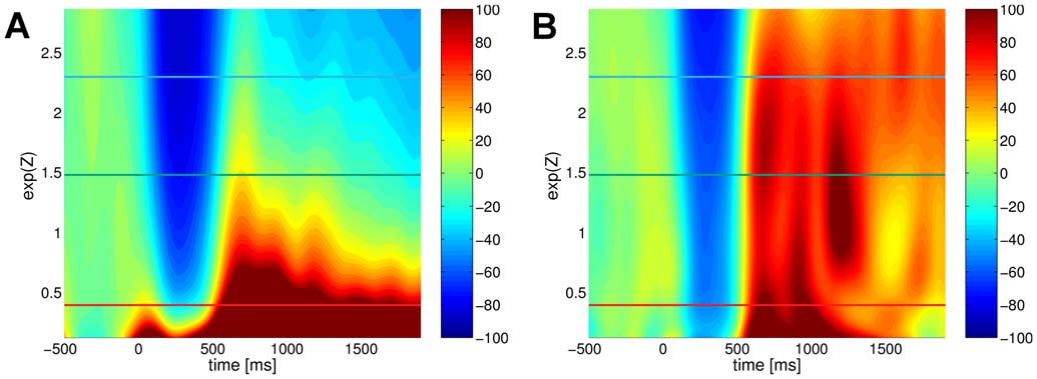
Comparision of both methods for estimating state conditional 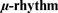 ERD induced by somatosensory stimuli. The figure contrasts the conventional (panel A) with the generalized state conditional ERD (panel B). The vertical axis represents the explanatory factor, i.e., the level of pre-stimulus 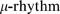 activity. The estimated ERD/S are depicted using a color coded scheme, where blue dyed areas correspond to periods of ERD while red dyed areas indicate an ERS. The additionally highlighted horizontal lines correspond to the individual panels of Fig. 6.

**Figure 6:**
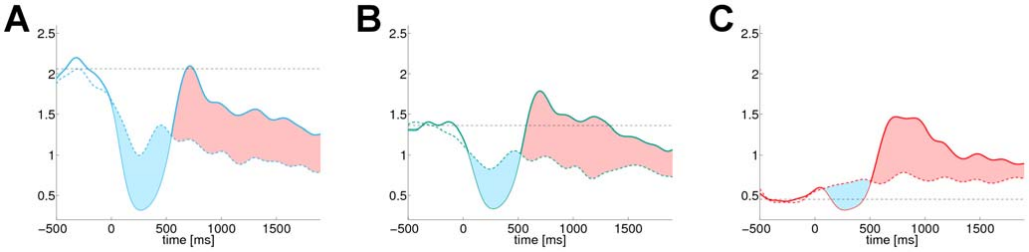
Estimated state conditional event-related and reference dynamics of the 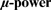. The individual panels A–C correspond to high, medium and low level of local pre-stimulus 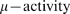. The dyed areas indicate the periods of ERD (blue) and ERS (red) identified by the generalized framework. The horizontal line in each panel illustrates the static baseline level of the conventional ERD, while the conditional event-related and reference dynamics are depicted by the solid and dashed dynamics, respectively.

Before presenting the results of the estimated interrelationship between the explanatory variables and the characteristics of the ERD response, we make a final comparison of the conventional and the generalized ERD framework. To this end, Fig. 5 gives an overview of the correspondingly estimated conventional and generalized state conditional ERD, in case of the 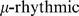 pre-stimulus activity 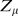 as the explanatory variable. Remarkably, the conventional ERD yields a rather variable result, analogous to the observations of spurious ERD/S for the artificial data sets whereas the generalized approach reveals that the brain reacts to the somatosensory stimulus in a highly systematic biphasic ERD-ERS sequence independent of the preceding state with either low or high amplitudes of ongoing 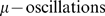. In order to further investigate this observation, we selected three representative states of the explanatory variable 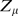 at high, medium and low activity (indicated by the horizontal lines in Fig. 5). At these states Fig. 6 depicts the estimated event-related dynamics along with the reference dynamics and the respective periods of ERD and ERS. The non-stationarity of the conditional reference dynamics is clearly detectable by their distinct linear trend. Those trends result mainly from the spontaneous spindle-like fluctuation of the 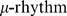. Specifically, periods of high activity (spindles) are likely to be followed of periods of lower activity, resulting in a negative trend of the conditional unperturbed dynamics and vice versa. This explains why in case of low pre-stimulus activity the conventional measure falsely yields an period of ERS without any preceding ERD. Moreover, for high activity preceding the stimulation the conventional measure returns an ERD period only. In strong contrast, the generalized ERD framework homogeneously reveals a standard biphasic ERD-ERS complex.

**Figure 7:**
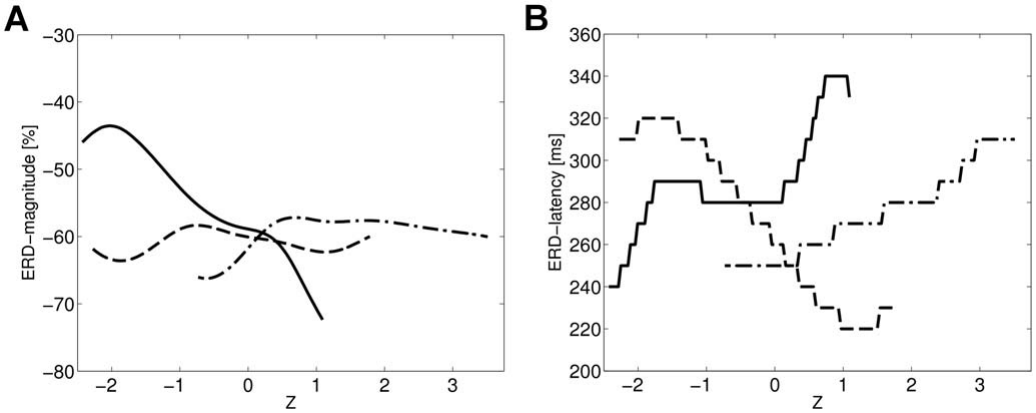
Estimated functional relationship between the ERD characteristics and the explanatory factors. Panel A shows the estimated dependency of ERD magnitude on the three explanatory factors, i.e., on the local pre-stimulus 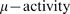 (solid), on the pre-stimulus activity of occipital 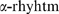 (dash-dotted) and on the strength of the preceding ERS response (dashed). The estimated relationship between ERD latency and the three factors is depicted in panel B.

For further investigations we restrict ourselves solely to the generalized state conditional ERD. Using the proposed state conditional measure, we test the hypothesis of a monotonic interrelationship between the three explanatory variables and the ERD magnitude on the one hand and the ERD latency on the other. To this end, we define the latency and the magnitude of the ERD as a function of the explanatory variable, based on the minimum in the interval 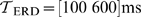:
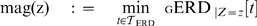
(21)

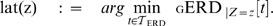
(22)In Fig. 7 the corresponding functions are illustrated for the three different explanatory variables. Note, the different domains of the state variables 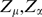 and 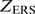. The step function like appearance in case of the ERD latency is due to sub-sampling the data to 100 Hz.

In order to quantitatively test for a monotonic interrelationship between the explanatory variable and the two dependent variables we used Spearman's rank correlation coefficient. The significance of a non-zero correlation coefficient was obtained by means of bootstrap confidence intervals, based on drawing 5000 bootstrap samples from the single trial data. For each bootstrap sample we separately estimated the generalized state conditional ERD along with the functional relationship between the ERD magnitude and latency and the three explanatory variables, yielding 5000 estimates of the Spearman's rank coefficients respectively. The particular estimation of the bootstrap confidence intervals for the correlation coefficients implemented the bias-correction and accelerated 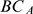 method introduced in [33]. We found a significant negative monotonic relationship between the magnitude of ERD and the local 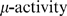 (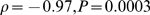), while the occipital 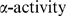 (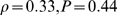) and the preceding ERS response (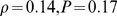) revealed no significant monotonic relationship. Physiologically, these results show that the ERD is stronger in case of immediately preceding higher pericentral mu-activity but independent from both, occipital alpha and local mu-activity some 2 second in the past; taken together, mu amplitude dynamics is a strictly local phenomenon, both in time and space. On the other hand, the latency of the ERD response showed a positive monotonic relationship with occipital 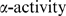 (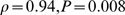); Thus, a lower occipital alpha, possibly indicative of system-wide increase of arousal, is reflected by a faster ERD at pericentral cortices. A negative monotonic relationship with the preceding ERS response strength(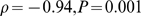) could indicate a persistent locally increased reactivity with fast ERDs after already strong responses to the last stimulus. No monotonic relationship was found for the 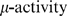 (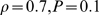).

## Discussion

We presented the novel data analytical framework of *gereralized ERD* that allows for a reliable analysis of ERD also in the presence of dynamical cortical states. To this end, we started from the observation that the conventional ERD measure can give rise to spurious detection of ERD, when analyzing non-stationary dynamics (Fig. 1-A). We then identified the constant baseline as the limiting factor of the conventional ERD measure. Accordingly, we generalized the conventional ERD framework with respect to the choice of reference. In particular, we substituted the constant baseline by a *reference dynamics* and derived a novel generalized measure for the quantification of ERD, by defining ERD/S as the relative deviation of the event-related dynamics from this reference dynamics. In particular, we proposed the use of the natural relaxation dynamics of the unperturbed EEG rhythm as a reference. In this context we also discussed how the acquisition of this reference dynamics can be incorporated into the experimental design by means of catch trials. Afterwards, we validated the ability of the generalized ERD measure to afford a reliable quantification of induced spectral perturbations even in the presence of non-stationary dynamics (Fig. 1-B). Moreover, we pointed out that the conventional and the generalized ERD measure yield identical results in case of stationary dynamics. Consequently, due to the lower effort in designing and conducting the experiment as well as in analyzing the data, if stationarity holds for the dynamics under study, then the conventional measure is preferred. However, we also emphasized, that stationarity cannot be assumed for investigations of state conditional dependencies.

Following the introduction of the generalized ERD framework, we extended both, the generalized and the conventional ERD measure in order to afford the quantification of *state conditional* ERD. Here, the application of a reliable state conditional measure can be used to scrutinize candidate explanatory factors, such as the level of activity of a particular EEG eigenrhythm or the stimulus intensity, with respect to their possible impact on a the oscillatory dynamics under study. As a proof of concept, we compared the respective capabilities of the conventional and the generalized state conditional framework first on simulated and afterwards on real ERD data. Here, in the well controlled scenario of artificially generated data, the comparison of the results of the conventionally estimated with the true analytically obtained state conditional ERD, clearly revealed the limitations of the conventional framework in retrieving the given functional relationship of the ERD on the explanatory variable. Furthermore, the conventional conditional ERD measure gave rise to spurious observations of ERD and ERS which were not even modelled in the artificial data (see Fig. 3). Unlike the conventional method, which failed, the novel generalized measures performed well at retrieving the true underlying functional relationship of the conditional ERD on an explanatory variable from the surrogate data (see Fig. 3). Finally, we illustrated the potential of the proposed novel framework for neurophysiological investigations by analyzing ERD data from a median nerve stimulation paradigm. In particular, we applied the novel estimator of generalized conditional ERD to analyze the impact of three explanatory factors on the inter-trial variability of the contra-lateral mu-rhythm ERD induced by somatosensory stimulations. Specifically, we investigated the impact of the magnitude of local prestimulus mu-rhythm activity, the magnitude of occipital alpha and the magnitude of the ERS response to the preceding stimulus on the ERD magnitude and latency. As a result, we found that the mu amplitude dynamics is a strictly local phenomenon, both in time and in space. Moreover, the application of the gereralized conditional ERD measure revealed that lower occipital alpha, possibly indicative of system-wide increase of arousal, can be linked to a faster mu rhythm ERD at pericentral cortices. Therefore, the proposed framework was able to provide evidence for the existence of a sensible physiological dynamics related to the interaction between ongoing activity and stimulus-induced responses.

In principle, the three given examples represent just a small sample of new possibilities: comparable analyses could be envisioned for the impact of various external factors such as: the inter-stimulus interval (ISI) [22], where short ISI results in stimulus presentation, while the processing of the previous event is still going on; the duration of the experiment, where the effects of fatigue on both, the event-related and the unperturbed dynamics can introduce variability of the ERD response; the simultaneous processing of multiple stimuli that potentially have a masking effect [34]; but also the influence of endogenous factors such as: the phase of a particular EEG eigenrhythm [17]; the synchronization level between adjacent cortical areas [35]; or causal coupling of various brain rhythms [36].

Moreover, recently the interest in inter-trial variability of ERD responses was sparked by the presentation of an alternative mechanism contributing to the generation of evoked responses [37]. In particular, the authors presented theoretical and empirical evidence that the amplitude fluctuations of neuronal alpha oscillations can be associated with changes in the mean value (baseline shift) of ongoing activity. Furthermore, they proved, when stimuli modulate the amplitude of alpha oscillations, these baseline shifts become the basis of a novel mechanism for the generation of evoked responses. Consequently, combining the two kinds of analysis, i.e., the analysis of ERD variability with the interpretation of ERD as a mechanism for the generation of ERP, may result in an additional explanation of inter-trial variability of ERPs.

Another important direct application area is brain-computer interfacing [38]–[40] which could benefit from this generalized conditional ERD framework: here, classifiers that discriminate between, e.g., imaginary left and right hand movements, could possibly yield an improved accuracy when considering state dependent behavior of ERD.

While there are a series of advantages and potentials, the application of the generalized framework comes at the expense of an experimental paradigm which has to comprise both, event-related and catch trials. Additional demands for a reliable estimation of state conditional ERD originate from the greater number of required trials compared to the estimation of unconditional ERD.

Notably, EEG scalp recordings mainly measure excitability fluctuations of superficial cortical layers, with minimal or no information on subcortical relays of the neural network supporting a given rhythm, e.g., an increased thalamic excitability may result in a low amplitude desynchronized cortical EEG [41]. Therefore, a cortical ERD is to be conceived as an electrophysiological index of an activated thalamo-cortical system involved in the processing of sensory or cognitive information or in the production of motor behavior [42]. While the analysis of cortico-subcortical interaction is naturally limited when based on scalp EEG data only, the modelling of inter-trial variability of evoked responses can improve the understanding of cortico-cortical interactions on a macroscopic scale [43] and it is here that the generalized conditional ERD represents a useful tool for such analyses with respect to accompanying ERD/S responses.

## Supporting Information

Text S1 - Supplementary Methods - In the Supplementary Methods file we present details on the empirical estimators for conditional ERD for both, discrete and continuous valued state variable. Moreover, details of the preprocessing applied to the somatosensory EEG data are given.
(0.19 MB PDF)
