# Supplementary material for: A Generalized Framework for Quantifying the Dynamics of EEG Event-Related Desynchronization
Source: PLoS Comput Biol. 2009 Aug 7;5(8):e1000453. doi: 10.1371/journal.pcbi.1000453 (PMC2713829; doi:10.1371/journal.pcbi.1000453)
Supplement: Text S1 — - Supplementary Methods - In the Supplementary Methods file we present details on the empirical estimators for conditional ERD for both, discrete and continuous valued state variable. Moreover, details of the preprocessing applied to the somatosensory EEG data are given. (0.19 MB PDF) [file pcbi.1000453.s001.pdf]

Supplementary Methods

Empirical Estimator for Conditonal ERD

Suppose a finite set of $2K$ observed single trials, i.e., $\mathcal{D} = \left\{\left(P_\cdot^k, c^k, z^k\right)\right\}_{k=1}^{2K}$, where $P_\cdot^k = (P_t)_{t\in\mathcal{T}}$ represents the instantaneous power of the rhythmic activity under investigation of the kth single trial in the interval $\mathcal{T}$. Correspondingly z^k denotes the realizations of the explanatory variable, while c^k serves as the indicator variable, that distinguishes between catch and event-related trials. For simplicity we will assume an equal number of K observed trials at both conditions and denote the corresponding subsets of trials by $\mathcal{C}^0 = \{k : c^k = 0\}$ and $\mathcal{C}^1 = \{k : c^k = 1\}$, respectively. In order to attain an empirical estimator for

$$\text{gERD}_{\,|Z=z}[t] := \frac{\mathbb{E}[P_t\,|C=1, Z=z]}{\mathbb{E}[P_t\,|C=0, Z=z]} - 1, \quad t \in \mathcal{T}, \tag{1}$$

the generalized conditional ERD, we have to find the corresponding empirical estimators for the conditional expectations. In case of a discrete variable Z the empirical estimators are easy to derive as straightforward average across trials with identical realization $Z = z$. Using $\mathcal{C}^i(z) := \{k \in C^i : z^k = z\}, i = 0, 1$ for the set of trials with realization $Z = z$, the empirical estimators of the expectation values in (1) become

$$\mathbb{E}_{emp}[P_t\,|C=i, Z=z, \mathcal{D}] = \frac{1}{|\mathcal{C}^i(z)|} \sum_{k\in\mathcal{C}^i(z)} P_t^k, \qquad i = 0, 1. \tag{2}$$

A continuously valued variable Z requires a more extended approach: For each instance $t \in \mathcal{T}$, the factorization theorem of probability theory ensures the existence of functions $\psi_t^0, \psi_t^1 : \mathbb{R} \to \mathbb{R}$, such that

$$\psi_t^0(z) = \mathbb{E}[P_t\,|Z=z, C=0] \quad \text{and} \quad \psi_t^1(z) = \mathbb{E}[P_t\,|Z=z, C=1]\,, \tag{3}$$

where for each instance $t \in \mathcal{T}$, ψ_t^0 and ψ_t^1 correspond to a separate regression function of the explanatory variable Z on the dependent variable P_t for the rest and the event-related condition, respectively. In principle any regression model could be fitted to the observed data $\mathcal{D}$. However, since the regression model has to be repeatedly estimated at every instance t, we propose the use a Nadaraya Watson estimator [1, 2] for reasons of efficiency. In case of $\psi_t^1(z)$ the Nadaraya Watson estimator is of the following form

$$\hat{\psi}_t^1(z\,|\mathcal{D}) = \sum_{k\in\mathcal{C}^1} P_t^k\, g_k^1(z), \tag{4}$$

where the local weighting factors $g_k^1(z)$ are given as

$$g_k^1(z) = \Phi\left(\frac{z^k - z}{h}\right)\left(\sum_{l\in\mathcal{C}^1} \Phi\left(\frac{z^l - z}{h}\right)\right)^{-1}. \tag{5}$$

Those weightings depend on the choice of the kernel function Φ as well as on the local smoothing parameter h. In the present analysis we used Gaussian kernels along with the optimal (with respect to the Asymptotic Mean Integrated Squared Error) choice of the bandwidth [3], i.e.,

$$h := \left(\frac{4}{3}\right)^{\frac{1}{5}} \sigma_Z \, K^{-\frac{1}{5}} \approx 1.06 \, \hat{\sigma}_Z \, K^{-\frac{1}{5}}, \tag{6}$$

where σ_Z is the standard deviation of the distribution of Z, $\hat{\sigma}_Z$ the corresponding empirical estimate and K the number of observations. Advantageously, the weighting factors $g^1_k(z)$ are independent of t and can therefore be efficiently calculated in a single sweep directly from the realizations of the explanatory variable Z.

Accordingly, we define the empirical estimator $\hat{\psi}^0_t(z \,|\mathcal{D})$ of the state conditional dynamics at rest by just exchanging the set $\mathcal{C}^1$ with $\mathcal{C}^0$, such that the empirical estimator for *conditional gERD* is finally determined by

$$\text{gERD}_{\,|Z=z}[t \,|\mathcal{D}] = \frac{\hat{\psi}^1_t(z \,|\mathcal{D})}{\hat{\psi}^0_t(z \,|\mathcal{D})} - 1. \tag{7}$$

Remark: For the sake of completeness we would like to mention that the empirical estimator of the simple extension of the conventional framework towards state conditonal ERD, i.e.,

$$\text{ERD}_{\,|Z=z}[t] := \frac{\mathbb{E}[P_t \,|Z=z]}{\mathbb{E}[P_{\text{REF}} \,|Z=z]} - 1, \quad t \in \mathcal{T}, \tag{8}$$

can be attained in an identical manner. To this end the empirical estimator of the conditional event-related dynamics (4) has to be contrasted with an estimate of the conditional expectation of the static baseline level, where the latter can be derived (cf. above) as a weigthed average of the single trial observations of the baseline levels $\{P^k_{\text{REF}} : k \in \mathcal{C}^1\}$, that is strictly speaking

$$\hat{\psi}_{\text{REF}}(z \,|\mathcal{D}) = \sum_{k \in \mathcal{C}^1} P^k_{\text{REF}} \, g^1_k(z). \tag{9}$$

Data preprocessing

Spatial Projection Generally, EEG signals obtained at individual sensors are often composed as a (linear) superposition of several distinct signals. In order to recover the signals of interest, while simultaneously suppressing interferences, we applied a specialised method to derive optimal spatial linear filters. For the present analysis of *conditional ERD* two spatial filters are required, one for the left-hemispheric μ-rhythm and the other for occipital α-activity. To this end, we used the Common Spatial Pattern (CSP) algorithm [4] on the bandpass filtered signal to project onto the signal originating from the contra-lateral somatosensory cortex, while an Independent Component Analysis (ICA) was applied on the broadband signals for the extraction of an occipital α-source.

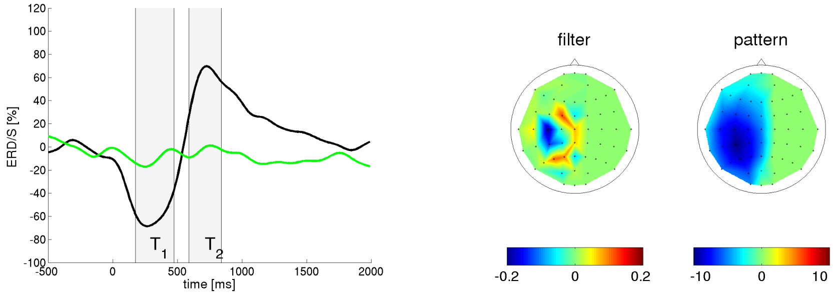

Figure 1: The left panel shows the conventionally estimated ERD response to the second stimulus, separately for the stimulation condition (black) and the reference condition (green). The virtual conditions T_1 and T_2 for the CSP are indicated by the gray shaded regions. The color panels at the right depict the CSP filter and the spatial distribution of the recovered source.

In general, the CSP analysis solves the task of finding a linear subspace, i.e., a linear combination of channels, for which the variance of the signal is maximized for one condition while the variance of another condition is minimized. This concept can be efficiently used to recover neural sources that exhibit ERD and ERS effects [5]. To this end we defined two virtual conditions based on the observed *averaged ERD* from an arbitrary sensor over the left somatosensory cortex. The first condition was defined as the ERD period, while the opposing condition is set to the ERS period (cf. left panel in Fig. 1). Applying the CSP algorithm to the bandpass (10 Hz) filtered signal determines an optimal spatial filter that maximizes the variance (the power) during the ERS period while it is minimized for the ERD phase. Intuitively, the derived spatial filter reflects the best linear spatial projection onto the modulated rhythmic μ-activity. The estimated spatial filter along with the corresponding scalp distribution of the recovered source are depicted in Fig. 1.

Occipital α-activity was extracted by means of an ICA algorithm that was applied to the broadband signals. For the present application we used the Temporal Decorrelation SEParation (TDSEP) algorithm [6], which exclusively relies on second-order statistics in the form of temporally delayed covariance matrices. Fig. 2 depicts the estimated spatial filter along with the scalp distribution of the extracted occipital α-source.

Time-Frequency Representation. Elaborated analyses of the temporal evolution of evoked spectral perturbations require a high-resolution representation of the data in the time-frequency domain. We used Morlet wavelets, which are known to achieve the best ratio between the resolution in the time and in the frequency domain. Moreover, Morlet wavelets are complex valued filters, which give rise to analytic signals and thereby enable access to the instantaneous phase and the instantaneous amplitude of rhythmic activity. For an easy introduction to wavelet decomposition, with a particular emphasis on Morlet

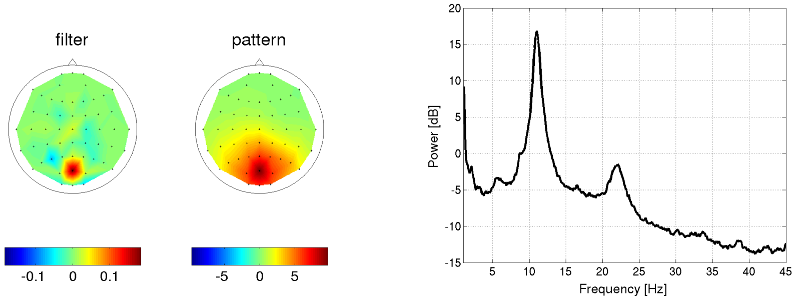

Figure 2: Left: The ICA-filter and the corresponding scalp distribution of the recovered occipital α source. The right panel shows the corresponding power spectrum with a distinct peak at 11 Hz.

wavelets we refer to [7]. In order to bandpass filter the EEG signals, we first determined the individual spectral peak in the 8–13 Hz domain at sensors covering the somatosensory and the occipital region congruently at 11 Hz. Secondly, we applied a Morlet wavelet centered at the individual spectral peaks.

Referring to the obtained spatial and spectral filters intuitively as $w_{\text{CSP}}, w_{\text{ICA}}$ and b_{11Hz}, respectively, we obtained the instantaneous power of contralateral μ-rhythm and occipital α-rhythm as:

$$P := \left| w_{\text{CSP}}^{\top} \cdot X * b_{\text{11Hz}} \right|^2 \qquad \text{and} \qquad O := \left| w_{\text{ICA}}^{\top} \cdot X * b_{\text{11Hz}} \right|^2, \tag{10}$$

where X represents the multi-channel single trial EEG.

References

[1] Nadaraya, E. On estimating regression. *Theory of Probability and its Applications* **10**, 186–90 (1964).

[2] Watson, G. Smooth regression analysis. *Shankya Series A* **26**, 359–72 (1964).

[3] Scott, D. *Multivariate Density Estimation: Theory, Practice, and Visualization* (John Wiley, New York, 1992).

[4] Fukunaga, K. *Introduction to Statistical Pattern Recognition* (Academic Press, New York, 1972).

[5] Blankertz, B., Tomioka, R., Lemm, S., Kawanabe, M. & Müller, K.-R. Optimizing spatial filters for robust EEG single-trial analysis. *IEEE Signal Proc Magazine* **25**, 41–56 (2008). URL `http://dx.doi.org/10.1109/MSP.2008.4408441`.

[6] Ziehe, A. & Müller, K.-R. TDSEP – an efficient algorithm for blind separation using time structure. In Niklasson, L., Bodén, M. & Ziemke, T. (eds.)

Proceedings of the 8th International Conference on Artificial Neural Networks, ICANN'98, Perspectives in Neural Computing, 675 – 680 (Springer Verlag, Berlin, 1998).

[7] Torrence, C. & Compo, G. A practical guide to wavelet analysis. *Bull. Am. Meterol.* **79**, 61–78 (1998).
